# Initial validation of the Turkish version of the defense mechanisms rating scales-self-report-30

**DOI:** 10.3389/fpsyg.2024.1432170

**Published:** 2024-06-26

**Authors:** Meltem Yılmaz, Berke Taş, Deniz Çelik, J. Christopher Perry, Annalisa Tanzilli, Mariagrazia Di Giuseppe, Vittorio Lingiardi

**Affiliations:** ^1^Department of Dynamic and Clinical Psychology, and Health Studies, Sapienza University of Rome, Rome, Italy; ^2^Department of Psychology, TED University, Ankara, Türkiye; ^3^Department of Psychology, Çankaya University, Ankara, Türkiye; ^4^Department of Psychiatry, McGill University and Institute of Community and Family Psychiatry at Jewish General Hospital, Montreal, QC, Canada; ^5^Department of History, Humanities and Society, University of Rome Tor Vergata, Rome, Italy

**Keywords:** defense mechanisms, defensive functioning, DMRS-SR-30, validation, psychometric properties

## Abstract

The Defense Mechanisms Rating Scales-Self Report-30 (DMRS-SR-30) was recently developed to add a self-report alternative to the assessment of defenses, reflecting their generally accepted hierarchical organization. In this study, we aimed to examine psychometric properties and factor structure of the Turkish language version of the DMRS-SR-30. The sample consisted of 1.002 participants who filled out a survey comprising the DMRS-SR-30, the Brief Symptom Inventory, and the Inventory of Personality Organization through Qualtrics. Confirmatory Factor Analysis indicated a three-factor structure (*CFI* = 0.89, *RMSEA* = 0.05) that confirms the DMRS theoretical frame with a relatively acceptable fit. Defensive categories and total scale scores showed good to excellent reliability (α values ranging from 0.64 to 0.89). Correlations between defenses, symptoms, and personality functioning demonstrated good convergent and discriminant validity. The individuals with clinically significant BSI scores (*T*-score ≥ 63) differed on the DMRS-SR-30 scores from the individuals in the non-clinical range. The Turkish version of the DMRS-SR-30 is a reliable and valid instrument to self-assess the hierarchy of defense mechanisms and overall defensive functioning. Moreover, the current study supports the validity of the tripartite model of defenses in a language and culture different from the origins of the DMRS and DMRS-SR-30.

## 1 Introduction

The concept of defense was initially conceived in psychoanalytic theory by Freud (1894), who suggested that defense mechanisms originate from unconscious conflicts and serve to protect the individual from the experience of intolerable, contradictory, and unacceptable feelings, ideas, memories, or wishes. Since these could not be consciously handled, they must be repressed and managed by symbolic emotional, cognitive, and behavioral processes known as defense mechanisms (Cramer, 1991; Perry, 2014). Empirical research has demonstrated that an individual can be partially or totally aware of certain defense mechanisms, particularly those higher in the hierarchy of adaptation (i.e., mature defenses) (Di Giuseppe et al., 2021; Békés et al., 2023). From the second half of the past century, the increased interest in empirical assessment of defense mechanisms has led to the development of several instruments, including self-report measures, observer-rated techniques, and projective tests (Cramer, 1991; Guldberg et al., 1993; Lerner, 2005). Despite the strengths and limitations of the different methods and measures, the main problem has been that defenses were evaluated differently by each measure which has hindered the comparison of research findings; this is reflected in the variety of defenses assessed, scoring systems, and factorial structures (see Silverman and Aafjes-van Doorn, 2023, for review). With the development of the Defense Mechanisms Rating Scales (DMRS; Perry, 1990), researchers and clinicians finally had a reliable method reflecting the hierarchical relationships of individual defenses to their general adaptiveness, which appears to be the closest candidate for a gold-standard for assessing defensive functioning (Di Giuseppe and Lingiardi, 2023). Following successful research using the DMRS (e.g., Perry and Cooper, 1989; Perry, 2001; Perry and Bond, 2012), other DMRS-based instruments were recently developed and validated (Berney et al., 2014; Di Giuseppe et al., 2014, 2020, 2021).

The DMRS has a distinguishing feature among other measures because it relies on the hierarchy of defenses first popularized by Vaillant (1971, 1992) and later enriched and operationalized in the observer-rated DMRS manual (Perry, 1990, 2014). The DMRS inspired the inclusion of a provisional Axis for defense mechanisms assessment in the fourth edition of the Diagnostic and Statistical Manual of Mental Disorders (DSM-IV; American Psychiatric Association, 1994). The DMRS hierarchical organization of defenses includes 30 defense mechanisms, sorted into three defensive categories (from most to least adaptive): mature (or high adaptive), neurotic, and immature. They are further ordered into seven defense levels (from the most to the least adaptive): high-adaptive, obsessional, neurotic, minor image-distorting, disavowal, major image-distorting, and action, each of which is characterized by a specific defensive function (see Perry, 2014, for review). The DMRS detailed scoring system is also derived from this theoretical and empirical model. It provides scores for the Overall Defensive Functioning (ODF), an overall index of defensive maturity or adaptiveness, as well as percentage scores for each category, defense level, and single defense mechanism, for a total of 41 subscale scores.

A large body of literature has applied the DMRS in research and has demonstrated that defense mechanisms are strongly related to mental health (Perry and Cooper, 1989; Perry and Bond, 2012; Di Giuseppe et al., 2022; Perry et al., 2022; Carlucci et al., 2023). Mature defensive functioning is associated with better personality functioning and fewer symptoms (Giovanardi et al., 2021; Tanzilli et al., 2022; Conversano et al., 2023; Martino et al., 2023; Messina et al., 2023), while immature defensive functioning is related to psychopathology (Perry and Cooper, 1989; Babl et al., 2019; Boldrini et al., 2020; Conversano et al., 2020; Carone et al., 2023; Vierl et al., 2023). Severe personality disorders are associated with low defensive functioning, action defenses, and major image-distorting defenses, which predict depression, suicidal attempts, substance use, and self-harming behavior (Perry and Cooper, 1989; Perry et al., 2020; Fiorentino et al., 2024). Thus, defense mechanisms constitute an important aspect of the personality organization (Maffei et al., 1995; Euler et al., 2019). For instance, anaclitic personality configuration, depicted as preoccupation with establishing satisfying interpersonal relationships, was found to be related to avoidance-focused defenses including denial, repression, and displacement. On the other hand, introjective personality organization, defined as the distorted overemphasis on maintaining a stable and positive self-identity, is revealed to be associated with the use of counter-active defenses such as projection and acting out (Cramer and Blatt, 1993). Furthermore, it has been shown that histrionic-narcissistic patients commonly use minor image distorting and neurotic defenses, while histrionic-borderline patients mainly use action and major image-distortion defenses (Lingiardi et al., 1999).

In the Turkish context, the Defense Mechanisms Inventory was the first validated and translated measure, but assessed only five defense mechanisms (Ihilevic and Gleser, 1995; Sorias et al., 1995). Due to its limited number of assessed defenses, the DMI is not widely used in clinical settings (Yılmaz et al., 2007). Later on, the Defense Style Questionnaire (DSQ; Bond et al., 1983; Bodur, 1999) and its shorter versions (DSQ-40; Andrews et al., 1993; Yılmaz et al., 2007) were validated in the Turkish language. The DSQ originally identified four hierarchically ordered defense styles (i.e., maladaptive, image-distorting, self-sacrificing, and adaptive defense styles), which have been only partially confirmed in later validation studies. Moreover, the DSQ has shown potentially problematic psychometric properties in some studies, such as low internal consistency for adaptive style (*r* = 0.57) and low test–retest reliability for image-distorting style (*r* = 0.63) (San Martini et al., 2004). Poor face validity (e.g., inability to correctly identify items corresponding to specific defenses by expert raters), and unstable factor structure were also found in the 40-item short form (Chabrol et al., 2005; Prout et al., 2018). In particular, the Turkish version of the DSQ was reported to have inconsistent factor structure and low discriminating validity in differentiating general from clinical populations (Bodur, 1999). Similarly, as in the DSQ long version, the Neurotic subscale (alpha = 0.61) of the DSQ-40 Turkish version was on the edge of the acceptable limit for coefficient alpha (alpha = 0.60) (Yılmaz et al., 2007).

The lack of a valid and reliable measure to assess defenses in the Turkish-speaking population inspired our research group to translate the Defense Mechanisms Rating Scales-Self-Report-30 (DMRS-SR-30; Di Giuseppe et al., 2020). This is a 30-item questionnaire developed from the DMRS (Perry, 1990) which assesses the whole hierarchy of defenses in both general and clinical populations (see Supplementary Figure 1S). Our aim is to examine the psychometric properties of the Turkish version of the questionnaire. The DMRS-SR-30 evaluates 28 defense mechanisms, whose reported factorial structure reflects the largely documented tripartite hierarchical organization of defenses (Prout et al., 2022). This measure has been proved to have excellent to good reliability for the ODF and defensive categories, as well as good convergent, divergent, criterion, and concurrent validity for both the Italian (Di Giuseppe et al., 2020) and the English (Prout et al., 2022) versions of the scale. Specifically, we aimed to investigate (1) the internal consistency for the ODF and defensive categories; (2) the convergent and discriminant validity; and (3) the factor structure of the Turkish version of the DMRS-SR-30 (Appendix).

As for the first research question, we expected to confirm good to excellent values for Cronbach’s alphas (Di Giuseppe et al., 2020; Prout et al., 2022). Concerning the second research question, we expected to confirm good convergent and discriminant validity of the Turkish version of the DMRS-SR-30 by examining the correlations with the Brief Symptom Inventory (BSI; Derogatis, 1993) and the Inventory of Personality Organization (IPO; Lenzenweger et al., 2001), and by examining the discriminating power of the DMRS-SR-30 between non-clinical and clinical groups formed based on the cut-off score of the BSI. Finally, for the third research question, we expected to replicate the hierarchically organized tripartite model of defenses developed based on the DMRS theory via Confirmatory Factor Analysis in a Turkish sample (Perry, 1990; Perry, 2014).

## 2 Methods

### 2.1 Participants

Participants were reached via social media, email listservs, and the dean’s office of several universities in Türkiye [hide for peer review]. Data collection was performed via Qualtrics between April 15, 2022 and June 2, 2023. Out of 1.852 participants who provided informed consent, 1.123 individuals filled out the online survey with less than 10 missing values in each measure and were included in the study. Missing values were handled at two steps: first, participants who did not fill more than 10 items of each assessment tool were deleted; second, the linear trend at point method was undertaken to replace the missing values by their predicted values (Meyers et al., 2016). As shown in previous research, participants who did not have at least seven items with non-zero score in the DMRS-SR-30 tend to have a score of 100% in one specific defense level which indicates poor rating pattern, since it is theoretically not possible to use only one defense level but not others (Perry et al., 2022). Five participants were erased from the data accordingly. To meet the assumptions of the statistical analyses, multivariate outliers were investigated with Mahalanobis distance (Meyers et al., 2016) and 116 participants were deleted from the data. Following these steps, the final data comprised 1.002 participants.

The sample (*N* = 1.002) primarily consisted of participants who were female (*N* = 833; 83.13%), heterosexual (87%, *N* = 852), and with a university degree (*N* = 792; 79.04%). The mean age of the sample (*N* = 996) was 37.65 (SD = 12.75) with a range of 18 to 75.

Further information on the demographic characteristics of the sample including the means and standard deviations for the Brief Symptom Inventory and the Inventory of Personality Organization could be found in the supplemental material (see Supplementary Tables 1S, 2S). The data that support the findings of this study are available from the corresponding author upon reasonable request.

### 2.2 Measures

#### 2.2.1 Socio-demographic variables

A socio-demographic questionnaire gathered data on the participant’s sex, gender, age, level of education, occupation, economic status, marital and relationship status, the existence of current and past psychological difficulties, and treatment histories.

#### 2.2.2 Defense mechanisms rating scales-self report-30

The DMRS-SR-30 is a self-report questionnaire consisting of 30 items taken from the DMRS and the DMRS Q-Sort version (DMRS-Q; Di Giuseppe et al., 2014, 2020; available online at; https://webapp.dmrs-q.com). It assesses the use of 28 hierarchically organized defense mechanisms with each item rated on a 5-point Likert scale, from not at all (0) to very often/much (4). Two defenses, passive aggression and dissociation, are each assessed by 2 items, whereas two defenses, devaluation and idealization, have their respective self and other items combined. The DMRS-SR-30 provides the 28 individual scores reflecting the percentage of all defenses attributable to each defense. The percentages are combined into the 7 hierarchical defense levels, and then 3 overall summary categories (mature, neurotic and immature, with the latter divided into depressive and non-depressive defenses) (see Di Giuseppe and Perry, 2021 for review). Total sum scores below 8 were found to be related with outliers, such as having one defense mechanism accounting for 50% of a participant’s ODF, or with a score of either 1 (lowest) or 7 (highest) (Di Giuseppe et al., 2020). These three components are dimensional and add up to 100, representing the percentage of each component’s use by the patient. These seven components are dimensional and add up to 100.

The DMRS-SR-30 has been validated in English and Italian languages. It demonstrated good reliability for ODF and defensive categories, ranging from 0.68 to 0.89, and good criterion (varying from 0.59 to 0.77) and concurrent validity (varying from 0.27 to 0.63) in comparison to both the DMRS and the DMRS-Q (Di Giuseppe et al., 2020). Good convergent and discriminant validity were found with measures assessing psychological symptoms and traumatic experiences in childhood. A three-factor structure was identified, reflecting the theoretical distribution of defenses within mature, neurotic, and immature defensive categories (Prout et al., 2022).

#### 2.2.3 Brief symptom inventory

The BSI is a 53-item self-report questionnaire derived from the original longer version of Symptom Checklist-90-Revised (SCL-90-R; Derogatis, 1975, 1977, 1993). It assesses psychological symptoms experienced by the individual during the past week. Items are scored on a 5-point Likert scale from 0 (not at all) to 4 (extremely) (Derogatis et al., 1973). In addition to an overall Global Symptom Inventory (GSI) score, there are 9 symptom scales [see list in Table 2]. Based on the dimension scores, which are obtained by dividing the sum of the values for items pertaining to a particular dimension by the number of items included in that dimension, T scores of 63 or above in GSI could be considered clinical cases. The BSI has been subject to extensive psychometric evaluation analyses (Derogatis, 1983, 1993), including its reliability and validity analyses in the Turkish language, and demonstrated adequate psychometric properties (Şahin and Durak, 1994; Şahin et al., 2002).

#### 2.2.4 The inventory of personality organization

The IPO is a 57-item assessment tool aiming to evaluate constructs related to Kernberg’s theory of personality organization on a 5-point Likert scale ranging from never true (1) to always true (5) (Kernberg, 1975; Kernberg and Clarkin, 1995; Lenzenweger et al., 2001). The IPO provides scores for three subscales: reality testing (i.e., ability to discriminate between self and non-self sources of stimuli in line with conventional norms of reality), identity diffusion (i.e., incoherent and defective self and other representations which prevent the integration of identity), and primitive defenses (i.e., immature defense mechanisms including splitting, projection, idealization, and devaluation). The IPO has adequate psychometric properties demonstrated by various studies conducted in both clinical and non-clinical individuals (Lenzenweger et al., 2001; Berghuis et al., 2009). Turkish adaptation of the inventory also presented high internal consistency, sufficient test–retest reliability along with robust construct and criterion validity (Ceran Yıldırım et al., 2020). Since items with factor loadings below 0.30 and with correlations less than 0.50 in the anti-image matrix were removed from the inventory, the final Turkish version of IPO contains 31 items (9 items for primitive defenses, α = 0.77; 10 items for identity diffusion, α = 0.82; 12 items for reality testing, α = 0.85).

### 2.3 Procedures

Prior to implementing the study, we translated all items from English to Turkish (forward translation), which was followed by the modifications and reconciliation of item wordings (forward translation reconciliation). An independent translator without prior knowledge of the DMRS-SR-30, back-translated the Turkish scale to English (back translation), then we translated English items back to Turkish again to reach the final and current version of the questionnaire. The entire procedure (translation, back translation, and item revision) was supervised by the authors of the original DMRS-SR-30 and an independent researcher.

Every procedure was carried out in accordance with institutional policies and applicable regulations, and it was authorized by the relevant institutional body. Following the ethical approval from two local Institutional Review Boards [hide for peer review], the online data was collected by sharing the Qualtrics link in social media (e.g., Facebook, Twitter, Instagram), email groups and with the dean’s office of above listed universities. At the beginning of the online survey, participants were presented with the aims and content of the study and were asked to sign an informed consent. They continued the survey by answering a list of demographic questions and by filling three randomly-distributed questionnaires (i.e., DMRS-SR-30, IPO, BSI). The average time required to complete the survey was 23 min. This study’s design and its analysis were not preregistered.

### 2.4 Statistical analysis

All the statistical analysis except for CFA was conducted with IBM SPSS Statistics, Version 26 (IBM Corp, 2019). Descriptive statistics, Cronbach’s alpha and Composite Reliability (CR) were examined for ODF, defensive categories, and defense levels to test internal consistency. Since Cronbach’s alpha is unweighted (i.e., indicators are assumed to be equally reliable) and assumes linearity (i.e., a linear relationship between each item and the total score), it is recommended to report CR (i.e., the total amount of true score variance in relation to the total scale score variance), which is a weighted estimate of reliability that does not assume error terms to be uncorrelated (Fornell and Larcker, 1981). For both Cronbach’s alpha and CR, the recommended benchmark value is 0.70, and any value above 0.60 is considered acceptable (Fornell and Larcker, 1981; Cicchetti, 1994; Hair et al., 2020).

Due to the non-normal distributions of the scales, we applied square-root transformations to the variables included in the analysis as it transformed the current data closest to normal distribution compared to other transformation methods (Meyers et al., 2016). For convergent and discriminant validity, Spearman’s rho correlations (*r*_s_) between the DMRS-SR-30 and both the BSI and the IPO were calculated on the transformed data to better control for non-normality in certain subscales which remained slightly deviated from normal distribution after data transformation. The interpretation of *r*_s_ is similar to the interpretation of Pearson’s correlation coefficients, as values above 0.70 considered strong and values between 0.40 and 0.70 moderate, which are expected to be above 0.30 to establish convergent validity (Akoglu, 2018).

Additionally, we also checked the Average Variance Extracted (AVE) based on the standardized factor loadings obtained from CFA described below to further establish convergent validity in combination with CR. For AVE, 0.50 is considered acceptable (Hair et al., 2020), and if AVE is less than 0.50 with a CR value higher than 0.60, the convergent validity of the construct could be claimed adequate (Fornell and Larcker, 1981).

Moreover, six separate Analysis of Covariance (ANCOVA) were conducted to establish further discriminant validity based on the DMRS-SR-30’s capacity to discriminate clinical-level symptomatology. The dependent variables for these analyses were ODF, mature, neurotic, immature, non-depressive and depressive defense categories. The independent variable was “the group” which was created based on the BSI cut-off T-score of 63 (i.e., non-clinical group, *T* < 63 vs. clinical group, *T* ≥ 63) (Derogatis and Melisaratos, 1983). For each of the analyses, age, sex and education were controlled.

In order to substantiate the tripartite hierarchical model of defense mechanisms based on the original categorization from the DMRS prior to the development of the DMRS-30-SR (Perry, 2014) and largely replicated by Prout et al. (2022), Confirmatory Factor Analysis (CFA) with the maximum likelihood estimation and robust standard error calculation was conducted on JASP 0.16.4 (JASP Team, 2023). Proposed residual covariances between the items belonging to the same factor were added to the model since items of a defense category are theoretically associated with each other besides statistical recommendations on their covariances (Cole et al., 2007). For the Comparative Fit Index (CFI), literature proposes ≥0.95 as an excellent fit and ≥ 0.90 as an acceptable fit (Hu and Bentler, 1999; McDonald and Ho, 2002; Kline, 2005). The indices of the Root Mean Square Error of Approximation (RMSEA) ≤ 0.06 and the Standardized Root Mean Square Residual (SRMR) ≤ 0.09 were suggested to indicate good fit (Hu and Bentler, 1999; Hooper et al., 2008).

## 3 Results

### 3.1 Internal consistency for the ODF, defensive categories, defense levels, and DMRS-SR-30 factors

Internal consistency was assessed with both Cronbach’s alpha (α) and Composite Reliability (CR) as displayed in Table 1. Based on the cut-off proposed by Cicchetti (1994) for alpha and Fornell and Larcker (1981) for CR, ODF and both the neurotic and immature defensive categories demonstrated adequate to excellent internal consistency. The mature category, the most prevalent defensive category in our sample (*M* = 41.76%, *SD* = 11.76) had an acceptable alpha and CR value (α = 0.64; CR = 0.65). Internal consistency of the Defense Levels ranged between 0.52 and 0.70, considered sufficient to good.

**Table 1:** Internal consistency of the Defense Mechanism Rating Scales Self-Report (DMRS-SR-30) based on overall defensive functioning (ODF), defensive categories, and defense levels (*N* = 1,002).

	α	CR	Mean (SD)	No of items	Range
ODF	0.90	0.92	5.09 (0.48)	30	3.73–6.75
Defensive categories					
Mature	0.64	0.65	41.76 (11.76)	8	15.38–87.27
Neurotic	0.77	0.77	23.88 (6.20)	8	0–43.14
Immature	0.86	0.85	34.37 (10.34)	14	0–72.73
Non-depressive	0.62	0.62	13.17 (5.32)	5	0–36.36
Depressive	0.82	0.80	21.20 (8.03)	9	0–48.89
Defense levels					
High Adaptive	0.64	0.65	41.76 (11.76)	8	15.38–87.27
Obsessional	0.52	0.53	9.03 (3.99)	3	0–24.24
Neurotic	0.70	0.70	14.86 (4.70)	5	0–38.46
Minor I-D	0.57	0.50	7.54 (4.11)	3	0–21.74
Disavowal	0.63	0.64	11.74 (5.01)	4	0–52.38
Major I-D	0.60	0.60	7.45 (4.26)	3	0–22.22
Action	0.70	0.60	7.65 (3.95)	4	0–28.95

I-D, Image Distortion; SD, standard deviation; α, Cronbach’s alpha; CR, composite reliability.

### 3.2 Convergent and discriminant validity

Based on AVE (ODF = 0.28; mature = 0.21; neurotic = 0.31; immature = 0.30) and CR estimates (see Table 1), the convergent validity of the constructs “defensive functioning”, “mature defensive category”, “neurotic defensive category”, and “immature defensive category” could be claimed adequate (Fornell and Larcker, 1981).

Moreover, convergent and discriminant validity of the DMRS-SR-30 ODF, defensive categories, and defense levels with the BSI and the IPO based on Spearman’s rho correlations are displayed in Table 2, 3.

**Table 2:** Convergent and discriminant validity of the DMRS-SR-30 based on its correlations with the brief symptom inventory (*N* = 1,002).

	SOM	OC	IS	DEP	ANX	HOS	PHOB	PAR	PSY	BSI total
ODF	−0.34**	−0.38**	−0.52**	−0.48**	−0.47**	−0.52**	−0.36**	−0.52**	−0.50**	−0.55**
*Defensive categories*										
Mature	−0.39**	−0.49**	−0.54**	−0.53**	−0.52**	−0.52**	−0.41**	−0.52**	−0.55**	−0.60**
Neurotic	0.27**	0.38**	0.19**	0.27**	0.28**	0.18**	0.17**	0.11**	0.25**	0.31**
Immature	0.27**	0.31**	0.47**	0.41**	0.40**	0.46**	0.33**	0.50**	0.44**	0.48**
Non-Depressive	0.12**	0.18**	0.19**	0.18**	0.15**	0.19**	0.15**	0.22**	0.21**	0.22**
Depressive	0.26**	0.28**	0.47**	0.41**	0.41**	0.47**	0.32**	0.49**	0.42**	0.47**
*Defense levels*										
High adaptive	−0.39**	−0.49**	−0.54**	−0.53**	−0.52**	−0.52**	−0.41**	−0.53**	−0.55**	−0.60**
Obsessional	0.13**	0.25**	0.10**	0.17**	0.16**	0.15**	0.15**	0.10**	0.18**	0.20**
Neurotic	0.25**	0.30**	0.18**	0.23**	0.25**	0.12**	0.11**	0.09**	0.19**	0.26**
Minor I-D	0.05	0.08**	0.15**	0.12**	0.08**	0.15**	0.13**	0.22**	0.17**	0.15**
Disavowal	0.20**	0.28**	0.31**	0.29**	0.26**	0.25**	0.23**	0.31**	0.28**	0.33**
Major I-D	0.23**	0.27**	0.41**	0.39**	0.36**	0.35**	0.27**	0.40**	0.38**	0.41**
Action	0.16**	0.12**	0.24**	0.19**	0.23**	0.36**	0.16**	0.26**	0.22**	0.25**

I-D, Image distortion; SOM, Somatization; OC, Obsession-compulsion; IS, Interpersonal sensitivity; DEP, Depression; ANX, Anxiety; HOS, Hostility; PHOB, Phobic anxiety; PAR, Paranoid ideation; PSY, Psychoticism; **p < 0.001.

**Table 3:** Convergent and discriminant validity of the DMRS-SR-30 based on its correlations with the Inventory of Personality Organization (IPO) (*N* = 1,002).

	Reality testing	Identity diffusion	Primitive defenses	IPO total
ODF	−0.37**	−0.42**	−0.44**	−0.48**
*Defensive categories*				
Mature	−0.38**	−0.50**	−0.50**	−0.55**
Neurotic^a^	0.08**	0.25**	0.19**	0.23**
Immature	0.36**	0.39**	0.41**	0.45**
Non-Depressive	0.29**	0.24**	0.26**	0.30**
Depressive	0.29**	0.34**	0.37**	0.39**
*Defense levels*				
7 High adaptive	−0.38**	−0.50**	−0.50**	−0.55**
6 Obsessional	0.12**	0.19**	0.14**	0.19**
5 Neurotic^b^	−0.003	0.16**	0.12**	0.13**
4 Minor I-D	0.21**	0.16**	0.21**	0.21**
3 Disavowal	0.24**	0.33**	0.30**	0.35**
2 Major I-D	0.25**	0.32**	0.33**	0.36**
1 Action	0.22**	0.13**	0.16**	0.17**

I-D, Image distortion, ^a^ = neurotic defensive categories includes defense levels 6 (obsessional) and 5 (neurotic); ^b^ = neurotic defense level includes (level 5) includes individual defenses of repression, dissociation, reaction formation, and displacement; **p < 0.01 level.

All DMRS-SR-30 subscales were significantly associated with BSI symptoms subscales and BSI total scores (see Table 2). The ODF score was negatively correlated with all symptom groups. Among defensive categories, immature defenses showed the strongest positive relationship with the BSI total score, whereas mature defenses showed the only negative relationship. Regarding defense levels, high adaptive defense level was negatively associated with all symptom groups, while neurotic and immature defense levels were positively associated with all symptom groups. All symptom groups had associations of lower magnitude with neurotic defenses than with immature defenses except for somatization. Significant correlations between the DMRS-SR-30 defense categories and levels and the BSI subscales were all in the expected directions, thus demonstrating good convergent validity, ranging from 0.10 to 0.61 (Krabbe, 2017).

Regarding the correlations with the IPO subscales (see Table 3), all correlations were significant and followed the expected directions. The only exception was the correlation between neurotic defense level and reality testing, which resulted as non-significant. The positive correlations between the IPO subscales and the DMRS-SR-30 neurotic and immature defense categories were between 0.12 to 0.55, indicating low to moderate correlations.

Finally, six separate ANCOVAs while controlling age, gender, and education level were conducted to compare non-clinical (*N* = 872) and clinical (*N* = 124) groups based on the BSI scores on defensive categories and ODF scores (see Table 4). Results suggested that clinical group scored significantly higher on neurotic [*F* (1, 991) = 19.01, *p* < 0.001] and immature [*F* (1, 991) = 54.46, *p* < 0.001] defense categories and, within the immature category, both depressive [*F* (1, 991) = 57.23, *p* < 0.001] and non-depressive [*F* (1, 991) = 9.97, *p* = 0.002] defensive subcategories compare to the non-clinical group. Moreover, clinical group scores were significantly lower on the mature defense category [*F* (1, 991) = 108.76, *p* < 0.001] and ODF score [*F* (1, 991) = 90.10, *p* < 0.001] than the non-clinical group. Overall, the ODF and three defense category scores successfully differentiate non-clinical and clinical groups in the expected direction.

**Table 4:** Descriptive statistics for defense categories and ODF scores based on BSI Groups (*N* = 996).

BSI groups	Non-clinical (*N* = 872)		Clinical (*N* = 124)	
	Mean	SD	Mean	SD
ODF	5.14 (2.48)	0.48 (0.10)	4.70 (2.39)	0.28 (0.6)
*Defensive categories*				
Mature	43.17 (6.59)	11.7 (0.85)	31.71 (5.70)	5.92 (0.52)
Neurotic	23.53 (4.90)	6.34 (0.73)	26.43 (5.22)	4.28 (0.41)
Immature	33.32 (5.77)	10.4 (1.02)	41.86 (8.53)	6.21 (0.49)
Depressive	20.39 (4.51)	7.94 (1.02)	27.12 (5.27)	5.78 (0.60)
Non-depressive	12.93 (3.64)	5.46 (0.83)	14.74 (3.94)	3.8 (0.51)

SD, standard deviation. Six participants were excluded from ANCOVAs since they did not report their age. Means and standard deviations reported in parentheses were calculated based on square root transformed data, however, to make the interpretation of the results easier, non-transformed means and SDs are reported in the table.

### 3.3 Factor structure based on confirmatory factor analysis

A relatively acceptable fit between the hypothesized model and the observed data was found (*χ*^2^(372) = 1273.21, *p* < 0.001, *χ*^2^/df = 3.422) with comparative fit index (CFI) = 0.889, Tucker-Lewis index (TLI) = 0.871, Root Mean Squared Error of Approximation (RMSEA) = 0.049, Standardized Root Mean Squared Residual (SRMS) = 0.049. Considering the complexity of the hierarchical model, when all fit indices are examined collectively, the model fit indices could be interpreted as a statistically acceptable model.

To provide a comprehensive view of the factor loadings, both standardized and unstandardized estimates are displayed in Table 5. Standardized factor loadings for immature defenses range from 0.29 (omnipotence) to 0.68 (devaluation), for neurotic defenses range from 0.39 (intellectualization) to 0.68 (dissociation), and for mature defenses range from 0.22 (sublimation) to 0.68 (self-observation), respectively. Although the factor loadings were all in line with expected magnitude, there were three exceptions which were slightly lower than the conventional value of 0.30 (Kline, 2005): sublimation (λ = 0.22), self-assertion (λ = 0.27) and omnipotence (λ = 0.29). Covariances between factors are displayed in Table 6.

**Table 5:** Factor structure of the DMRS-SR-30 based on confirmatory factor analysis (*N* = 1,002).

				95% CI		
Factor	Indicator	Estimate	Std. Est.	SE	Lower	Upper
Mature	Self-observation	0.636	0.676	0.032	0.573	0.698
	Suppression	0.632	0.595	0.043	0.548	0.716
	Anticipation	0.571	0.540	0.037	0.499	0.644
	Affiliation	0.466	0.443	0.038	0.391	0.540
	Humor	0.398	0.361	0.038	0.324	0.473
	Altruism	0.342	0.335	0.038	0.268	0.416
	Self-assertion	0.263	0.272	0.036	0.192	0.334
	Sublimation	0.249	0.216	0.045	0.161	0.336
Neurotic	Dissociation	0.829	0.682	0.032	0.765	0.892
	Undoing	0.726	0.676	0.030	0.667	0.786
	Dissociation	0.712	0.584	0.034	0.645	0.779
	Reaction formation	0.638	0.524	0.037	0.566	0.711
	Displacement	0.548	0.507	0.033	0.483	0.613
	Isolation of affect	0.507	0.505	0.033	0.443	0.571
	Repression	0.555	0.501	0.033	0.490	0.621
	Intellectualization	0.425	0.390	0.036	0.355	0.494
Immature	Devaluation	0.762	0.682	0.031	0.702	0.823
	Help-rejecting complaining	0.718	0.639	0.029	0.661	0.776
	splitting of self	0.724	0.638	0.033	0.659	0.789
	Rationalization	0.586	0.577	0.033	0.521	0.650
	Autistic fantasy	0.693	0.565	0.036	0.622	0.764
	Splitting of others	0.575	0.545	0.033	0.510	0.640
	Projection	0.595	0.539	0.034	0.528	0.663
	Projective identification	0.579	0.539	0.034	0.513	0.645
	Denial	0.546	0.535	0.031	0.485	0.608
	Passive aggression	0.531	0.531	0.035	0.462	0.600
	Idealization	0.563	0.522	0.033	0.499	0.627
	Passive aggression	0.454	0.474	0.032	0.391	0.516
	Acting out	0.421	0.430	0.034	0.355	0.487
	Omnipotence	0.265	0.289	0.033	0.201	0.330

SE, standard error, Std. Est., standardized estimate; CI, confidence interval.

**Table 6:** Factor covariances of the DMRS-SR-30 (*N* = 1,002).

						95% CI		
			Estimate	SE	*z*-value	*p*	Lower	Upper
Immature	↔	Neurotic	0.97	0.01	95.013	<0.001	0.95	0.99
Neurotic	↔	Mature	0.77	0.02	33.244	<0.001	0.73	0.82
Immature	↔	Mature	0.64	0.03	24.701	<0.001	0.59	0.70

SE, standard error; CI, confidence interval.

## 4 Discussion

Contemporary research literature suggests that multi-method assessment of personality constructs is essential to capture the different components of personality organization (Lenzenweger et al., 2001; Hopwood and Bornstein, 2014). In line with this rationale, following the gold standard evaluation of defenses by observer-rated scales (i.e., the DMRS and the DMRS-Q), the DMRS-SR-30 was developed to provide a valid and reliable measure to assess the hierarchy of defense mechanisms (Perry, 1990; Di Giuseppe and Lingiardi, 2023). The present validation study of the Turkish version of the DMRS-SR-30 revealed good to excellent psychometric properties and a factor structure almost fully confirming previous validations of the measure (Di Giuseppe et al., 2020; Prout et al., 2022).

The Turkish version of the DMRS-SR-30 demonstrated excellent internal consistency on the ODF, the two categories of neurotic and immature defenses, and an acceptable value for mature defenses. Similar reliability has been found for the three extracted factors in previous studies, which also largely matched the validated tripartite defensive categories model (Vaillant et al., 1986). Defense levels also showed acceptable internal consistency (ranging from 0.52 to 0.70). These results demonstrate that despite the low number of items included in each subscale, the DMRS-SR-30 reliability remains acceptable for defense levels, as demonstrated in previous validation studies (Di Giuseppe et al., 2020; Prout et al., 2022).

Regarding convergent and discriminant validation, correlations between the DMRS-SR-30 summary scales (i.e., ODF, defensive categories, defense levels) and the BSI subscales were significant and in expected directions. Participants with higher defensive functioning and greater use of mature defenses also showed significantly lower symptom scores as compared to participants with lower defensive functioning. Conversely, lower ODF and greater endorsement of neurotic and immature defenses were significantly positively related to all domains of psychological distress. Similar findings emerged when comparing the DMRS-SR-30 with the IPO subscales. As expected, immature defense levels were found to be associated with lower reality testing, whereas high-adaptive defense level was linked with higher capability in reality testing. An unexpected lack of association between neurotic defense level and reality testing should be further investigated. Overall, correlations were found in line with expected magnitude and directions, demonstrating good convergent and discriminant validity of the Turkish version of the DMRS-SR-30. Additionally, these findings confirmed previous evidence of the link between defenses and symptomatology (Perry and Cooper, 1989; Drapeau et al., 2011; Porcerelli et al., 2011) and of the relationship between defenses and personality functioning (Kernberg, 2006; Perry et al., 2013; Gagnon et al., 2016).

All symptom groups had associations of lower magnitude with neurotic defenses then with immature defenses except for somatization. There exists an enduring psychoanalytic assumption that bodily sensations could stem from the redirection or transfer of psychic energy toward physical symptoms. Focusing on the body might help individuals sidestep overwhelming emotions, acting as a shield against hostile thoughts as they perceive themselves physically injured, rather than devaluing or idealizing one’s own or others’ image as in minor-image distorting defenses (Busch, 2014; Rosa et al., 2019). This assumption was empirically investigated in various studies which shows that neurotic defenses such as displacement (Hyphantis et al., 2013) and undoing (Noorbala et al., 2018) are associated with somatic complaints (Sardella et al., 2022). The findings of this study showed that, regarding defensive categories, individuals most frequently reported using mature defenses, followed by immature defenses, with neurotic defenses being the least used, which is consistent with the literature (Békés et al., 2023).

With regard to the factor structure of the DMRS-SR-30 Turkish version, we largely confirm our hypothesis that the three factors would represent the tripartite hierarchy of defenses. We found a fit to the theoretical tripartite model closer to the acceptable level. This needs further confirmation using larger and more heterogeneous samples, including clinical populations.

Our findings should be interpreted in light of several limitations. First, online data collection might have restricted selection to individuals who have access to technology and have sufficient computer skills. Although online data collection is a frequently used method for gathering samples, future studies might benefit from applying in-person, pen-and-pencil data gathering. Second, our sample largely consisted of individuals recruited from the general population. As evidenced by the fact that 872 (87%) of our sample had a BSI score below the clinical cut-off, we had a limited inclusion of individuals with clinical level psychopathology. This restricted range on the BSI most likely reduced the magnitude of the relationship between defenses and symptoms that were found in clinical samples, thus affecting the generalizability of our findings. An example would be the relatively lower correlations found between action defense level and symptoms compared to our expectations based on prior theoretical and empirical studies. As found in previous research, individuals with low symptom severity usually endorse fewer action defenses since they are more prone to reflect on their inner conflicts and regulate their emotions instead of acting them out, or counter-attacking the external source to manage the immediate source of conflict (Perry et al., 2013; Perry, 2014). In this line, we expected higher correlations between action defense level and symptoms. Future studies should include a larger number of participants with greater variety of psychological conditions, which would provide a more adequate test of this. In sum, while age, profession, and marital status showed considerable diversity, limited variability in sex, sexual orientation, monthly income, education and the BSI scores might limit the generalizability of the findings to a sample primarily consisted of healthy females self-identified as heterosexual with university degrees having low to middle income who have adequate means to access the internet.

Third, only self-report measures were used in this study, which leaves unaddressed how defenses would be related to observer-based measures of symptoms and functioning. Future studies should include other observer-rated DMRS-based measures for a test of criterion validity; as well as inclusions of observer-rated assessments of symptoms and functioning to examine the utility or limitation of the self-report assessment of defenses (Di Giuseppe et al., 2020). A final point regards the risks that translating self-report statements from English to Turkish may yield a true equivalence of the words, and yet the meanings may have subtle differences in the second language. This could result in the same statement being associated with slightly different levels of adaptation across the two cultures.

In summary, the evaluation of defense mechanisms should consider the entire hierarchy of defenses assessed with valid and reliable tools. For decades, research on defense mechanisms has been limited by the absence of consent theoretical and empirical models, but thanks to the DMRS model and the new tools derived from it, it is now possible to assess defenses form a widely validated and effective gold standard perspective (Silverman and Aafjes-van Doorn, 2023).

## 5 Conclusion

The assessment of the hierarchy of defenses and the tripartite model has proven clinically useful, and the development of a self-report method makes this more accessible to research and clinical work. The present study provides a solid psychometric validation of a Turkish version of the DMRS-SR-30 extending its use in Turkish contexts. A theoretically and empirically grounded self-report questionnaire now can be employed by both clinicians and researchers in Türkiye to conduct research on psychopathology and psychotherapy process and outcome (see Gelo et al., 2015; Gelo and Manzo, 2015; Schiepek et al., 2020). Identifying defense mechanisms of patients for case formulation and tracking them throughout the therapy process with other patient and therapist factors is of utmost importance to create effective interventions and to identify factors that bring positive changes (Perry and Bond, 2012; Di Giuseppe et al., 2020). The availability of a short self-report assessment should facilitate the process of data collection, especially for the repetitive measurement of defenses and defensive functioning after therapy sessions. Moreover, this validated Turkish version of the DMRS-SR-30 will allow us to expand our knowledge on defensive organization in non-western cultures and to conduct further cross-cultural studies.

## Data availability statement

The raw data supporting the conclusions of this article will be made available by the authors on request, without undue reservation.

## Ethics statement

The current study was approved by the Scientific Research and Publication Ethics Committees of two universities: Istanbul Bilgi University, Türkiye (approval number: 2022-40024-59/25.03.2022), and Ankara Çankaya University, Türkiye (approval number: E-90705970-605-120544/11.01.2023). The studies were conducted in accordance with the local legislation and institutional requirements. The participants provided informed consent to participate by clicking a button in the online form.

## Author contributions

MY: Writing – review & editing, Writing – original draft, Project administration, Methodology, Investigation, Formal analysis, Data curation, Conceptualization. BT: Funding acquisition, Writing – review & editing, Writing – original draft, Project administration, Investigation, Formal analysis, Data curation. DC: Funding acquisition, Writing – review & editing, Writing – original draft, Project administration, Investigation, Formal analysis, Data curation. JP: Funding acquisition, Writing – review & editing, Validation, Supervision. AT: Resources, Funding acquisition, Writing – review & editing. MG: Validation, Methodology, Conceptualization, Writing – review & editing, Writing – original draft, Supervision. VL: Writing – review & editing, Writing – original draft, Validation, Supervision, Methodology, Conceptualization.

## References

[ref1] Akoglu H. (2018). User's guide to correlation coefficients. Turk. J. Emerg. Med. 18, 91–93. doi: 10.1016/j.tjem.2018.08.001, PMID: 30191186 PMC6107969

[ref2] American Psychiatric Association (1994). Diagnostic and statistical manual of mental disorders. 4th Edn. Washington, DC: American Psychiatric Press.

[ref3] Andrews G. Singh M. Bond M. (1993). The defense style questionnaire. J. Nerv. Ment. Dis. 181, 246–256. doi: 10.1097/00005053-199304000-00006 8473876

[ref4] Babl A. Grosse Holtforth M. Perry J. C. Schneider N. Dommann E. Heer S.. (2019). Comparison and change of defense mechanisms over the course of psychotherapy in patients with depression or anxiety disorder: evidence from a randomized controlled trial. J. Affect. Disord. 252, 212–220. doi: 10.1016/j.jad.2019.04.021 30986736

[ref5] Békés V. Perry J. C. Starrs C. J. Prout T. A. Conversano C. Di Giuseppe M. (2023). Defense mechanisms are associated with mental health symptoms across six countries. Res. Psychother. Psychopathol. Process Outcome 26. doi: 10.4081/ripppo.2023.729, PMID: 38226792 PMC10849071

[ref6] Berghuis H. Kamphuis J. H. Boedijn G. Verheul R. (2009). Psychometric properties and validity of the Dutch inventory of personality organization (IPO-NL). Bull. Menn. Clin. 73, 44–60. doi: 10.1521/bumc.2009.73.1.44, PMID: 19413469

[ref7] Berney S. de Roten Y. Beretta V. Kramer U. Despland J. N. (2014). Identifying psychotic defenses in a clinical interview. J. Clin. Psychol. In Session 70, 428–439. doi: 10.1002/jclp.22087 24691714

[ref8] Bodur F. (1999). Ego Savunma Mekanizmaları testinin Türkçe formu dil eşdeğerliliği, güvenirlik ve geçerlilik çalışması. Master’s thesis, İstanbul University: Counsil of Higher Education (CoHE) Thesis Center.

[ref9] Boldrini T. Tanzilli A. Di Cicilia G. Gualco I. Lingiardi V. Salcuni S.. (2020). Personality traits and disorders in adolescents at clinical high risk for psychosis: toward a clinically meaningful diagnosis. Front. Psych. 11, 1–11. doi: 10.3389/fpsyt.2020.562835, PMID: 33363479 PMC7753018

[ref10] Bond M. Gardner S. T. Christian J. (1983). Empirical study of self-rated defense styles. Arch. Gen. Psychiatry 40, 333–338. doi: 10.1001/archpsyc.1983.01790030103013, PMID: 6830412

[ref11] Busch F. N. (2014). Clinical approaches to somatization. J. Clin. Psychol. 70, 419–427. doi: 10.1002/jclp.22086 24677173

[ref12] Carlucci S. Chyurlia L. Presniak M. Mcquaid N. Wiebe S. Hill R.. (2023). Assessing defense mechanisms in binge-eating disorder: preliminary validity and reliability of the defense mechanism rating scale (DMRS) coded from adult attachment interviews. Psychoanal. Psychol. 40, 279–287. doi: 10.1037/pap0000457

[ref13] Carone N. Benzi I. M. A. Muzi L. Parolin L. A. L. Fontana A. (2023). Problematic internet use in emerging adulthood to escape from maternal helicopter parenting: defensive functioning as a mediating mechanism. Res. Psychother. Psychopathol. Process Outcome 26. doi: 10.4081/ripppo.2023.693, PMID: 37946531 PMC10715189

[ref14] Chabrol H. Rousseau A. Rodgers R. Callahan S. Pirlot G. Sztulman H. (2005). A study of the face validity of the 40 item version of the defense style questionnaire (DSQ-40). J. Nerv. Ment. Dis. 193, 756–758. doi: 10.1097/01.nmd.0000185869.07322.ed, PMID: 16260933

[ref15] Cicchetti D. V. (1994). Guidelines, criteria, and rules of thumb for evaluating normed and standardized assessment instruments in psychology. Psychol. Assess. 6, 284–290. doi: 10.1037/1040-3590.6.4.284

[ref16] Cole D. A. Ciesla J. A. Steiger J. H. (2007). The insidious effects of failing to include design-driven correlated residuals in latent-variable covariance structure analysis. Psychol. Methods 12, 381–398. doi: 10.1037/1082-989X.12.4.381, PMID: 18179350

[ref17] Conversano C. Di Giuseppe M. Lingiardi V. (2023). Case report: changes in defense mechanisms, personality functioning, and body mass index during psychotherapy with patients with anorexia nervosa. Front. Psychol. 14, 1–7. doi: 10.3389/fpsyg.2023.1081467, PMID: 36895755 PMC9989464

[ref18] Conversano C. Di Giuseppe M. Miccoli M. Ciacchini R. Di Silvestre A. Sterzo R. L.. (2020). Retrospective analyses of psychological distress and defense style among cancer patients. Clin. Neuropsychiatry 17, 217–224. doi: 10.36131/cnfioritieditore20200403, PMID: 34908997 PMC8629055

[ref20] Cramer P. (1991). The development of defense mechanisms: Theory, research, and assessment. New York: Springer-Verlag.

[ref21] Cramer P. Blatt S. J. (1993). Change in defense mechanisms following intensive treatment, as related to personality organization and gender. Concept Def. Mech. Contemp. Psychol. 310-320. doi: 10.1007/978-1-4613-8303-1_21

[ref22] Derogatis L. R. (1975). The SCL-90-R. Baltimore, MD: Clinical Psychometric Research.

[ref23] Derogatis L. R. (1977). SCL-90-R: Administration scoring and procedures manual I. Baltimore, MD: Clinical Psychometric Research.

[ref24] Derogatis L. R. (1983). SCL-90-R: Administration, scoring & procedures manual-II for the revised version and other instruments of the psychopathology rating scale series.

[ref25] Derogatis L. R. (1993). The brief symptom inventory-BSI administration, scoring and procedures manual. 4th Edn. Minneapolis, MN: National Computer Systems.

[ref26] Derogatis L. R. Lipman R. Covi L. (1973). SCL-90: an outpatient psychiatric rating scale—preliminary report. Psychopharmacol. Bull. 9, 13–28, PMID: 4682398

[ref27] Derogatis L. R. Melisaratos N. (1983). The brief symptom inventory: an introductory report. Psychol. Med. 13, 595–605. doi: 10.1017/S0033291700048017, PMID: 6622612

[ref28] Di Giuseppe M. Lingiardi V. (2023). From theory to practice: the need of restyling definitions and assessment methodologies of coping and defense mechanisms. Clin. Psychol. Sci. Pract. 30, 393–395. doi: 10.1037/cps0000145

[ref29] Di Giuseppe M. Orrù G. Gemignani A. Ciacchini R. Miniati M. Conversano C. (2022). Mindfulness and defense mechanisms as explicit and implicit emotion regulation strategies against psychological distress during massive catastrophic events. Int. J. Environ. Res. Public Health 19, 1–10. doi: 10.3390/ijerph191912690, PMID: 36231993 PMC9566362

[ref30] Di Giuseppe M. Perry J. C. (2021). The hierarchy of defense mechanisms: assessing defensive functioning with the defense mechanisms rating scales Q-sort. Front. Psychol. 12:440. doi: 10.3389/fpsyg.2021.718440, PMID: 34721167 PMC8555762

[ref31] Di Giuseppe M. Perry J. C. Lucchesi M. Michelini M. Vitiello S. Piantanida A.. (2020). Preliminary validity and reliability of the novel self-report based on the defense mechanisms rating scales (DMRS-SR-30). Front. Psych. 11:870. doi: 10.3389/fpsyt.2020.00870, PMID: 33005160 PMC7479239

[ref32] Di Giuseppe M. Perry J. C. Petraglia J. Janzen J. Lingiardi V. (2014). Development of a Q-sort version of the defense mechanism rating scales (DMRS-Q) for clinical use. J. Clin. Psychol. 70, 452–465. doi: 10.1002/jclp.22089, PMID: 24706519

[ref33] Di Giuseppe M. Perry J. C. Prout T. A. Conversano C. (2021). Editorial: recent empirical research and methodologies in defense mechanisms: defenses as fundamental contributors to adaptation. Front. Psychol. 12:602. doi: 10.3389/fpsyg.2021.802602, PMID: 34925197 PMC8678405

[ref34] Drapeau M. de Roten Y. Blake E. Beretta V. Strack M. Körner A.. (2011). Defensive flexibility and its relation to symptom severity, depression, and anxiety. J. Nerv. Ment. Dis. 199, 38–41. doi: 10.1097/NMD.0b013e3182043b4e, PMID: 21206245

[ref35] Euler S. Stalujanis E. Allenbach G. Kolly S. de Roten Y. Despland J. N.. (2019). Dialectical behavior therapy skills training affects defense mechanisms in borderline personality disorder: an integrative approach of mechanisms in psychotherapy. Psychother. Res. J. Soc. Psychother. Res. 29, 1074–1085. doi: 10.1080/10503307.2018.1497214, PMID: 30005584

[ref36] Fiorentino F. Lo Buglio G. Morelli M. Chirumbolo A. Di Giuseppe M. Lingiardi V.. (2024). Defensive functioning in individuals with depressive disorders: a systematic review and meta-analysis. J. Affect. Disord. 357, 42–50. doi: 10.1016/j.jad.2024.04.091, PMID: 38663554

[ref37] Fornell C. Larcker D. F. (1981). Structural equation models with unobservable variables and measurement error: Algebra and statistics. J. Mark. Res. 18:382. doi: 10.2307/3150980

[ref38] Freud S. (1894). “The neuropsychoses of defense”, in the standard edition of the complete psychological works of Sigmund Freud (1893–1899) early psycho-analytic publications. London: The Hogarth Press.

[ref39] Gagnon J. Vintiloiu A. McDuff P. (2016). Do splitting and identity diffusion have respective contributions to borderline impulsive behaviors? Input from Kernberg’s model of personality. Psychoanal. Psychol. 33, 420–436. doi: 10.1037/pap0000052

[ref41] Gelo O. C. G. Manzo S. (2015). “Quantitative approaches to treatment process, change process, and process-outcome research” in Psychotherapy research: Foundations, process, and outcome. eds. O. C. G. Gelo, A. Pritz, and B. Rieken (Vienna: Springer-Verlag), 247–277.

[ref42] Gelo O. C. G. Pritz A. Rieken B. (2015). “Preface” in Psychotherapy research: Foundations, process, and outcome. eds. O. C. G. Gelo, A. Pritz, and B. Rieken (Vienna: Springer-Verlag), v–vi.

[ref43] Giovanardi G. Mirabella M. Di Giuseppe M. Lombardo F. Speranza A. M. Lingiardi V. (2021). Defensive functioning of individuals diagnosed with gender dysphoria at the beginning of their hormonal treatment. Front. Psychol. 12:665547. doi: 10.3389/fpsyg.2021.665547, PMID: 34484028 PMC8415164

[ref44] Guldberg C. A. Høglend P. Perry J. C. (1993). Scientific methods for assessing psychological defenses. Nord. J. Psychiatry 47, 435–446. doi: 10.3109/08039489309104112

[ref45] Hair J. F. Jr. Howard M. C. Nitzl C. (2020). Assessing measurement model quality in PLS-SEM using confirmatory composite analysis. J. Bus. Res. 109, 101–110. doi: 10.1016/j.jbusres.2019.11.069

[ref46] Hooper D. Coughlan J. Mullen M. R. (2008). Evaluating model fit: a synthesis of the structural equation modelling literature. Electron. J. Bus. Res. Methods 6, 53–60. doi: 10.21427/D79B73

[ref47] Hopwood C. J. Bornstein R. F. (2014). Multimethod clinical assessment. New York: Guilford Publications.

[ref48] Hu L. T. Bentler P. M. (1999). Cutoff criteria for fit indexes in covariance structure analysis: conventional criteria versus new alternatives. Struct. Equ. Model. Multidiscip. J. 6, 1–55. doi: 10.1080/10705519909540118

[ref49] Hyphantis T. Goulia P. Carvalho A. F. (2013). Personality traits, defense mechanisms and hostility features associated with somatic symptom severity in both health and disease. J. Psychosom. Res. 75, 362–369. doi: 10.1016/j.jpsychores.2013.08.014, PMID: 24119944

[ref50] IBM Corp (2019). IBM SPSS statistics for windows (version 26.0) [Computer software]. New York: IBM Corp.

[ref51] Ihilevic D. Gleser G. C. (1995). “Defense mechanisms inventory: its development and clinical applications. Ego defenses theory and measurement” in Comprehensive textbook of psychiatry. eds. Conte H. R. Plutchik R., 431–478.

[ref52] JASP Team (2023). JASP (version 0.17.3) [computer software]. Netherlands: JASP.

[ref53] Kernberg O. F. (1975). Borderline conditions and pathological narcissism. New York: Jason Aronson.

[ref54] Kernberg O. F. (2006). Identity: recent findings and clinical implications. Psychoanal. Q. 75, 969–1004. doi: 10.1002/j.2167-4086.2006.tb00065.x 17094369

[ref55] Kernberg O. F. Clarkin J. F. (1995). The inventory of personality organization. White Plains, NY: The New York Hospital-Cornell Medical Center.

[ref56] Kline T. J. B. (2005). Psychological testing: a practical approach to design and evaluation. London: Sage Publications.

[ref57] Krabbe P. F. M. (2017). The measurement of health and health status: concepts, methods and applications from a multidisciplinary perspective. London: Academic Press.

[ref58] Lenzenweger M. F. Clarkin J. F. Kernberg O. F. Foelsch P. A. (2001). The inventory of personality organization: psychometric properties, factorial composition, and criterion relations with affect, aggressive dyscontrol, psychosis proneness, and self-domains in a nonclinical sample. Psychol. Assess. 13, 577–591. doi: 10.1037/1040-3590.13.4.577, PMID: 11793901

[ref59] Lerner P. M. (2005). “Defense and its assessment: the Lerner defense scale” in Scoring the Rorschach: Seven validated systems. eds. Bornstein R. F. Masling J. M. (Mahwah, NJ: Lawrence Erlbaum Associates Publishers).

[ref60] Lingiardi V. Lonati C. Delucchi F. Fossati A. Vanzulli L. Maffei C. (1999). Defense mechanisms and personality disorders. J. Nerv. Ment. Dis. 187, 224–228. doi: 10.1097/00005053-199904000-00005 10221555

[ref61] Maffei C. Fossati A. Lingiardi V. Madeddu F. Borellini C. Petrachi M. (1995). Personality maladjustment, defenses, and psychopathological symptoms in nonclinical subjects. J. Personal. Disord. 9, 330–345. doi: 10.1521/pedi.1995.9.4.330

[ref62] Martino G. Catalano A. Viola A. Vicario C. M. Bellone F. Silvestro O.. (2023). Psychological impairment in inflammatory bowel diseases: the key role of coping and defense mechanisms. Res. Psychother. Psychopathol. Process Outcome 26. doi: 10.4081/ripppo.2023.731, PMID: 38224215 PMC10849073

[ref63] McDonald R. P. Ho M. H. R. (2002). Principles and practice in reporting structural equation analyses. Psychol. Methods 7, 64–82. doi: 10.1037/1082-989x.7.1.64, PMID: 11928891

[ref64] Messina I. Calvo V. Grecucci A. (2023). Attachment orientations and emotion regulation: new insights from the study of interpersonal emotion regulation strategies. Res. Psychother. Psychopathol. Process Outcome 26. doi: 10.4081/ripppo.2023.703, PMID: 38224213 PMC10849076

[ref65] Meyers L. S. Gamst G. Guarino A. J. (2016). Applied multivariate research: Design and interpretation. Thousand Oaks, California: Sage publications.

[ref66] Noorbala F. Ghorbani N. Lavasani M. (2018). Defense mechanisms and somatization: the effect of defense hierarchy on somatization. J. Appl. Psychol. Res. 9, 25–37.

[ref67] Perry J. C. (1990). Defense mechanism rating scales (DMRS). 5th Edn. Boston: The Cambridge Hospital.

[ref68] Perry J. C. (2001). A pilot study of defenses in adults with personality disorders entering psychotherapy. J. Nerv. Ment. Dis. 189, 651–660. doi: 10.1097/00005053-200110000-00001, PMID: 11708665

[ref69] Perry J. C. (2014). Anomalies and specific functions in the clinical identification of defense mechanisms: defensive anomalies in mental status. J. Clin. Psychol. 70, 406–418. doi: 10.1002/jclp.22085, PMID: 24677150

[ref70] Perry J. C. Banon E. Bond M. (2020). Change in defense mechanisms and depression in a pilot study of Antidepressive medications plus 20 sessions of psychotherapy for recurrent major depression. J. Nerv. Ment. Dis. 208, 261–268. doi: 10.1097/nmd.0000000000001112, PMID: 32221178

[ref71] Perry J. C. Bekes V. Starrs C. J. (2022). A systematic survey of adults' health-protective behavior use during early COVID-19 pandemic in Canada, Germany, United Kingdom, and the United States, and vaccination hesitancy and status eight months later. Prev. Med. Rep. 30, 1–8. doi: 10.1016/j.pmedr.2022.102013, PMID: 36246769 PMC9554196

[ref72] Perry J. C. Bond M. (2012). Change in defense mechanisms during long-term dynamic psychotherapy and five-year outcome. Am. J. Psychiatry 169, 916–925. doi: 10.1176/appi.ajp.2012.11091403, PMID: 22885667

[ref73] Perry J. C. Cooper S. H. (1989). An empirical study of defense mechanisms: I. Clinical interview and life vignette ratings. Arch. Gen. Psychiatry 46, 444–452. doi: 10.1001/archpsyc.1989.01810050058010 2712663

[ref74] Perry J. C. Presniak M. D. Olson T. R. (2013). Defense mechanisms in schizotypal, borderline, antisocial, and narcissistic personality disorders. Psychiatry 76, 32–52. doi: 10.1521/psyc.2013.76.1.32, PMID: 23458114

[ref75] Porcerelli J. H. Cogan R. Markova T. Miller K. Mickens L. (2011). The diagnostic and statistical manual of mental disorders, defensive functioning scale: a validity study. Compr. Psychiatry 52, 225–230. doi: 10.1016/j.comppsych.2010.06.003 21295230

[ref76] Prout T. A. Di Giuseppe M. Zilcha-Mano S. Perry J. C. Conversano C. (2022). Psychometric properties of the defense mechanisms rating scales-self-Report-30 (DMRS-SR-30): internal consistency, validity and factor structure. J. Pers. Assess. 104, 833–843. doi: 10.1080/00223891.2021.2019053, PMID: 35180013

[ref77] Prout T. A. Gottdiener W. H. Camargo A. Murphy S. (2018). The relationship between defense mechanisms and religious coping using a new two-factor solution for the defense style Questionnaire-40. Bull. Menn. Clin. 82, 224–252. doi: 10.1521/bumc.2018.82.3.224, PMID: 30179043

[ref78] Rosa V. Tomai M. Lauriola M. Martino G. Di Trani M. (2019). Body mass index, personality traits, and body image in Italian pre-adolescents: an opportunity for overweight prevention. Psihologija 52, 379–393. doi: 10.2298/PSI181121009R

[ref79] Şahin H. N. Batugün D. A. Uğurtaş S. (2002). Kısa Semptom Envanteri (KSE): Ergenler için kullanımının geçerlik, güvenilirlik ve faktör yapısı. Turk Psikiyatri Derg. 13, 125–135, PMID: 12794665

[ref80] Şahin N. H. Durak A. (1994). Kısa Semptom Envanteri: Türk gençleri için uyarlanması. Türk Psikoloji Dergisi 9, 44–56.

[ref81] San Martini P. Roma P. Sarti S. Lingiardi V. Bond M. (2004). Italian version of the defense style questionnaire. Compr. Psychiatry 45, 483–494. doi: 10.1016/j.comppsych.2004.07.012, PMID: 15526260

[ref82] Sardella A. Lenzo V. Basile G. Martino G. Quattropani M. C. (2022). Emotion regulation strategies and difficulties in older adults: a systematic review. Clin. Gerontol. J. Aging Ment. Health 46, 280–301. doi: 10.1080/07317115.2022.2128706, PMID: 36163629

[ref83] Schiepek G. Gelo O. Viol K. Kratzer L. Orsucci F. Felice G.. (2020). Complex individual pathways or standard tracks? A data-based discussion on the trajectories of change in psychotherapy. Counsell. Psychother. Res. 20, 689–702. doi: 10.1002/capr.12300

[ref84] Silverman J. Aafjes-van Doorn K. (2023). Coping and defense mechanisms: a scoping review. Clin. Psychol. Sci. Pract. 30, 381–392. doi: 10.1037/cps0000139

[ref85] Sorias O. Leblebici Ç. Uysal Ş. (1995). Savunma Mekanizmaları Envanteri’ni Türk kültürüne uyarlama çalışması. İzmir: Ege Üniversitesi.

[ref86] Tanzilli A. Cibelli A. Liotti M. Fiorentino F. Williams R. Lingiardi V. (2022). Personality, defenses, mentalization, and epistemic trust related to pandemic containment strategies and the COVID-19 vaccine: a sequential mediation model. Int. J. Environ. Res. Public Health 19, 1–22. doi: 10.3390/ijerph192114290, PMID: 36361183 PMC9656964

[ref87] Vaillant G. E. (1971). Theoretical hierarchy of adaptive ego mechanisms: a 30-year follow-up of 30 men selected for psychological health. Arch. Gen. Psychiatry 24, 107–118. doi: 10.1001/archpsyc.1971.01750080011003, PMID: 5539856

[ref88] Vaillant G. E. (1992). Ego mechanisms of defense: A guide for clinicians and researchers. Washington, DC: American Psychiatric Press.

[ref89] Vaillant G. E. Bond M. Vaillant C. O. (1986). An empirically validated hierarchy of defense mechanisms. Arch. Gen. Psychiatry 43, 786–794. doi: 10.1001/archpsyc.1986.01800080072010, PMID: 3729674

[ref90] Vierl L. Juen F. Benecke C. Hörz Sagstetter S. (2023). Exploring the associations between psychodynamic constructs and psychopathology: a network approach. Personal. Ment. Health 17, 40–54. doi: 10.1002/pmh.1559, PMID: 35879050

[ref91] Ceran Yıldırım G. G. Yüksel S. (2020). Kişilik organizasyonları envanterinin Türkçe uyarlaması: Geçerlik ve güvenirlik çalışması. Ulakbilge Sosyal Bilimler Dergisi 56, 1–19. doi: 10.7816/ulakbilge-09-56-01

[ref92] Yılmaz N. Gençöz T. Ak M. (2007). Savunma Biçimleri Testi'nin psikometrik özellikleri: Güvenilirlik ve geçerlik çalışması. Turk Psikiyatri Derg. 18, 244–253, PMID: 17853979

