# Supplementary material for: Initial validation of the Turkish version of the defense mechanisms rating scales-self-report-30
Source: Front Psychol. 2024 Jun 26;15:1432170. doi: 10.3389/fpsyg.2024.1432170 (PMC11233775; doi:10.3389/fpsyg.2024.1432170)
Supplement: Supplementary file 1 [file Table_1.DOCX]

Supplementary Material

**Initial validation of the Turkish version of the Defense Mechanisms Rating Scales-Self-Report-30 (DMRS-SR-30)**

**Meltem Yılmaz^1*^, Berke Taş^2^, Deniz Çelik^3^, J. Christopher Perry^4^, Annalisa Tanzilli^1^, Mariagrazia Di Giuseppe^5†^ & Vittorio Lingiardi^1†^**

***Correspondance:**

[**meltem.yilmaz@uniroma1.it**](mailto:meltem.yilmaz@uniroma1.it)

**Table 1S.** *Demographic Characteristics of the Sample* (*N*=1,002)

| **Variable** | ***N* (%)** |
| --- | --- |
| **Age**  (*N*=996; *M* = 37.65; *SD* = 12.75) |  |
| 18-24 | 149 (14.77) |
| 25-34 | 313 (31.24) |
| 35-44 | 262 (26.15) |
| 45-54 | 151 (15.07) |
| 55-64 | 103 (10.28) |
| 65-75 | 18 (1.80) |
| **Sex**  (*N*=1,001) |  |
| Female | 833 (83.13) |
| Male | 160 (15.97) |
| Other | 8 (0.80) |
| **Sexual Orientation**  (*N*=979) |  |
| Heterosexual | 852 (85.03) |
| Lesbian/Gay | 8 (0.80) |
| Bisexual | 33 (3.29) |
| Other/not tell | 86 (8.58) |
| **Education**  (*N*=1,002) |  |
| Primary School | 2 (0.20) |
| High School | 51 (5.09) |
| University student | 151 (15.07) |
| University Graduate | 442 (44.11) |
| Graduate student | 166 (16.57) |
| Graduate | 184 (18.36) |
| Other | 6 (0.60) |
| **Profession**  (*N*=1,002) |  |
| Unemployed | 52 (5.19) |
| Housewife | 75 (7.49) |
| Employee | 349 (34.83) |
| Retired | 94 (9.38) |
| Student | 192 (19.16) |
| Other | 240 (23.95) |
| **Monthly income ***  (*N*=987)  Below 11.000 TL  11.000-29.000 TL  Above 30.000 TL  **Marital Status**  (*N*=1,000) | 500 (50)  310 (31)  177 (17.7) |
| Not married | 441 (44.01) |
| Married | 522 (52.10) |
| Other | 37 (3.69) |
| **Relationship**  (*N*=988) |  |
| With Partner | 694 (69.26) |
| No Current Partner | 217 (21.66) |
| No History of Relationship | 77 (7.68) |
| **Psychological Problems**^  (*N* = 997) |  |
| Current + Past | 196 (20) |
| Current + No Past | 295 (29) |
| No Current + Past | 196 (20) |
| No Current + No Past | 310 (31) |

*Note.* *The minimum wage was 11.000 Turkish Liras at the time of the assessment; ^ The questions regarding current and past psychological problems were ‘Do you currently have any psychiatric/psychological problems or issues that challenge you mentally?’ and ‘In the past, did you have any psychiatric/psychological problems or issues that were mentally challenging for you, other than the ones you mentioned in the previous question?’

**Table 2S.** *Descriptive Statistics for the Brief Symptom Inventory and the Inventory of Personality Organization* (*N* =1,002)

|  | ***M*** | ***SD*** | **Range** | ***N* of items** |
| --- | --- | --- | --- | --- |
| **BSI** (*N* =1,002) |  |  |  |  |
| Total^ | 0.73 | 0.54 | 0-2.91 | 53 |
| SOM | 0.41 | 0.50 | 0-3.40 | 7 |
| OC | 1.04 | 0.75 | 0-4.00 | 6 |
| IS | 0.90 | 0.84 | 0-4.00 | 4 |
| Depression | 1.04 | 0.84 | 0-4.00 | 6 |
| Anxiety | 0.71 | 0.67 | 0-3.83 | 6 |
| Hostility | 0.67 | 0.67 | 0-4.00 | 5 |
| Phobic Anxiety | 0.35 | 0.49 | 0-3.60 | 5 |
| Paranoid Ideation | 0.89 | 0.71 | 0-4.00 | 5 |
| Psychotism | 0.50 | 0.53 | 0-3.40 | 5 |
| **IPO** (*N* =1,002) |  |  |  |  |
| Total | 50.60 | 12.55 | 0-99 | 31 |
| Reality testing | 15.32 | 3.41 | 0-36 | 12 |
| Identity diffusion | 20.69 | 6.34 | 0-41 | 10 |
| Primitive defenses | 14.59 | 4.67 | 0-35 | 9 |

*Note.* ODF = Overall Defensive Functioning; OC = Obsession-compulsion; IS = Interpersonal Sensitivity; ^Total score comprises additional 4 items which do not load under the listed factors but considered by the BSI developers as clinically important

**Figure 1S.** *The DMRS hierarchical organization of defensive categories, defense levels and individual defenses*


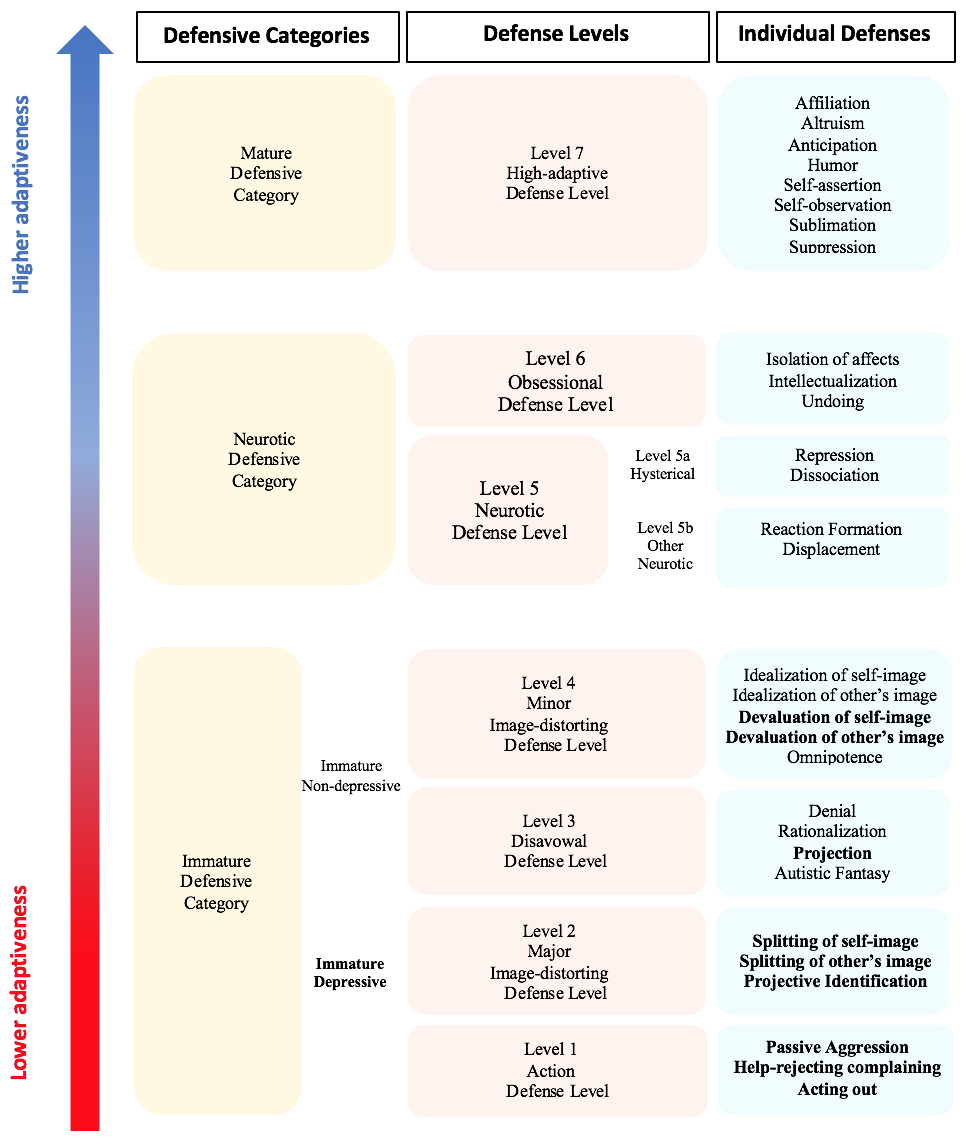
*Note.* Figure extracted from Di Giuseppe, M., & Perry, J. C. (2021). The Hierarchy of Defense Mechanisms: Assessing Defensive Functioning with the Defense Mechanisms Rating Scales Q-Sort. Frontiers in psychology, 12, 718440. <https://doi.org/10.3389/fpsyg.2021.718440>; Reprinted with permission. Depressive defenses are in bold.
