# Supplementary material for: Initial validation of the Turkish version of the defense mechanisms rating scales-self-report-30
Source: Front Psychol. 2024 Jun 26;15:1432170. doi: 10.3389/fpsyg.2024.1432170 (PMC11233775; doi:10.3389/fpsyg.2024.1432170)
Supplement: Supplementary file 2 [file Table_2.docx]

Supplementary Material

**Initial validation of the Turkish version of the Defense Mechanisms Rating Scales-Self-Report-30 (DMRS-SR-30)**

**Meltem Yılmaz^1*^, Berke Taş^2^, Deniz Çelik^3^, J. Christopher Perry^4^, Annalisa Tanzilli^1^, Mariagrazia Di Giuseppe^5†^ & Vittorio Lingiardi^1†^**

***Correspondance:**

[**meltem.yilmaz@uniroma1.it**](mailto:meltem.yilmaz@uniroma1.it)

**Appendix 1.** *The Turkish version of Defense Mechanisms Rating Scales-Self-Report-30 (DMRS-SR-30)*

*Savunma Mekanizmaları Değerlendirme Ölçeği-Özbildirim Formu-30 (SMDÖ-ÖF-30)*

| *Geçen hafta, sizi zorlayan duygular veya durumlarla başa çıkarken aşağıdaki yollardan hangilerini ne ölçüde kullandınız?* | |
| --- | --- |
| Hiç (0) Nadiren/Çok az (1) Bazen/Biraz (2) Sıklıkla/Çok (3) Çok sık/Fazla (4) | |
| 1. Başkalarını 'tamamen iyi' ya da 'tamamen kötü' olarak algıladınız mı? | 0 1 2 3 4 |
| 1. Kişisel olarak sizi ilgilendiren konulara karşı kayıtsız hissettiniz mi? | 0 1 2 3 4 |
| 1. Duygusal durumlar karşısında baş ağrısı, karın ağrısı, ya da herhangi bir işe başlarken zorlanma gibi fiziksel semptomlar geliştirdiniz mi? | 0 1 2 3 4 |
| 1. İhtiyacı olduğunu düşündüğünüz insanlara fiziksel veya psikolojik yardımda bulundunuz mu? | 0 1 2 3 4 |
| 1. Gerçek hayattan kaçıp içine çekildiğiniz, tekrarlayan ya da sürekli hayalleriniz oldu mu? | 0 1 2 3 4 |
| 1. İleride yaşayabileceğiniz muhtemel zorlukların üstesinden nasıl gelebileceğinizi düşüdünüz mü? | 0 1 2 3 4 |
| 1. Kendiniz hakkında hiç bir olumlu ya da toparlanabilir bir yan yokmuş gibi hissettiniz mi? | 0 1 2 3 4 |
| 1. İlişkilerinizde, aradaki farkı anlamadan, aldığınızdan daha çok verdiğinizi düşündürecek bir tutumunuz oldu mu? | 0 1 2 3 4 |
| 1. Bir problemle başa çıkmak için elinizden geleni yaptığınız sırada fiziksel veya duygusal destek talep ettiniz mi? | 0 1 2 3 4 |
| 1. Yaratıcı aktiviteler ile gerginliğinizi üzerinizden atmaya çalıştınız mı? | 0 1 2 3 4 |
| 1. Başkalarını güvenilmez veya manipülatif olarak algıladığınız ya da onlara süpheli bir tavır ile yaklaştığınız oldu mu? | 0 1 2 3 4 |
| 1. Sizi zorlayan kişisel meseleleri veya stresli durumları esprili yorumlar ile karşıladığınız oldu mu? | 0 1 2 3 4 |
| 1. Kişisel düşünceleriniz ve duygusal deneyimleriniz üzerine kafa yordunuz mu? | 0 1 2 3 4 |
| 1. Sinirinizi kendinizden çıkarıp, öfkenizi kendinize zarar vererek ifade ettiniz mi? | 0 1 2 3 4 |
| 1. Stresli durumların veya kişisel problemlerinizin asıl nedenlerinin üzerini örtmek için, mantıklı açıklamalar öne sürüp kendinizi haklı gösterdiğiniz oldu mu? | 0 1 2 3 4 |
| 1. Ortaya çıkan problemleri çözmek için aktif bir rol aldınız mı? | 0 1 2 3 4 |
| 1. Kendinizi ve/veya başkalarını kişisel özellikler üzerinden idealize ettiniz mi? | 0 1 2 3 4 |
| 1. Başkalarına, farkında olarak ya da olmayarak, tahrik edecek veya kızdıracak bir şekilde yaklaştınız mı? | 0 1 2 3 4 |
| 1. Yapılması gereken işleri halledebilmek için kişisel ihtiyaçlarınızı geçici olarak ertelediniz mi? | 0 1 2 3 4 |
| 1. Sizi endişelendiren bir problemden uzaklaşmak için dikkatinizi dağıtabilecek alakasız veya daha önemsiz meselelere odaklandığınız oldu mu? | 0 1 2 3 4 |
| 1. Duygusal bir konuyu, duygularınızı deneyimlemeden veya dikkate almadan, kendinizden uzaklaştırarak veya genel bir biçimde tartıştığınız oldu mu? | 0 1 2 3 4 |
| 1. Başkalarının sizi umursamayıp ilgilenmediğinden veya sizi anlamadığından şikayet ettiğiniz oldu mu? | 0 1 2 3 4 |
| 1. Birinin size kasti olarak hissettirmeye çalıştırdıklarını, sizin de o kişiye karşı yoğun olarak hissettiğiniz oldu mu? | 0 1 2 3 4 |
| 1. Sizi kaygılandıran bir konu hakkında konuşurken, kafanız karışık, boşluğa düşmüş veya üzerine konuşamaz bir halde hissettiğiniz oldu mu? | 0 1 2 3 4 |
| 1. Sözlü ve ya fiziksel bir kavgaya girdiniz mi? | 0 1 2 3 4 |
| 1. Akla getirmesi basit olan bazı şeyleri hatırlamakta zorlandığınız oldu mu? | 0 1 2 3 4 |
| 1. Kişisel problemler veya duygular hakkında düşünmekten kaçındınız mı? | 0 1 2 3 4 |
| 1. Kendinizi çok güçlü, üstün veya ulaşılamaz olarak hissettiniz mi? | 0 1 2 3 4 |
| 1. Kendinizi, sizi kaygılandıran bir konuda birbiriyle çelişen veya çatışmalı düşünceler içindeyken bulduğunuz oldu mu? | 0 1 2 3 4 |
| 1. Kendinizi ve/veya başkalarını kişisel özellikler yüzünden değersizleştirdiğiniz oldu mu? | 0 1 2 3 4 |
